# May–Thurner Syndrome: A Consideration for Deep Vein Thrombosis in Males

**DOI:** 10.1155/2020/2324637

**Published:** 2020-05-23

**Authors:** Tay Tian En Jason, Tay Jia Sheng, Tieng Chek Edward Choke, Pooja Sachdeva

**Affiliations:** ^1^Department of Internal Medicine, Singapore General Hospital, Singapore; ^2^Vascular and Endovascular Service, Department of General Surgery, Sengkang General Hospital, Singapore; ^3^Internal Medicine, Department of General Medicine, Sengkang General Hospital, Singapore

## Abstract

May–Thurner syndrome (MTS) is an underdiagnosed cause of lower limb deep vein thrombosis (DVT). The clinical prevalence of MTS-related DVT is likely underestimated, particularly in patients with other more recognisable risk factors. MTS is classically described in females between the age group of 20–50 years. In patients with acute iliofemoral thrombosis, medical treatment with anticoagulation alone has been associated with higher risk of postthrombotic syndrome (PTS) and lower iliofemoral patency rates, as compared to endovascular correction. We describe a case of MTS-related extensive iliofemoral DVT occurring in a middle age male who presented with acute onset of left lower limb swelling and pain, complicated by pulmonary embolism. Doppler compression ultrasonography of the left lower limb showed partial DVT extending from the left external iliac to the popliteal veins, and contrasted computed tomography (CT) of the thorax abdomen and pelvis established features of MTS, together with right pulmonary embolism. He was started on low molecular weight heparin (LMWH) and then underwent left lower limb AngioJet pharmacomechanical thrombolysis/thrombectomy, iliac vein stenting, and temporary inferior vena cava (IVC) filter insertion. After the procedure, the patient recovered and improved symptomatically with rapid resolution of this left lower limb swelling and pain. He was switched to an oral Factor Xa inhibitor and was subsequently discharged. After 1-month follow-up, he remained well with stent patency visualised on repeat ultrasound and underwent an uneventful elective IVC filter retrieval with subsequent plans for a 1-year follow-up.

## 1. Introduction

May–Thurner syndrome (MTS) was first described by May and Thurner in 1957 [1]. MTS is an anatomical variant in which the right common iliac artery overlies and compresses the left common iliac vein against the lumbar spine and is an important anatomical risk factor for venous thrombosis.

Although this variant has been shown to be present in more than 20% of the population [1, 2], the reported clinical prevalence of MTS-related deep vein thrombosis (DVT) has been low at only 2-3% of all lower limb DVTs [3, 4], suggesting an underestimation of its actual prevalence. The most common clinical presentation of MTS is unilateral leg swelling due to an acute DVT and is more common in women with the majority between 20 and 50 years of age [5]. Treatment options for MTS-related DVT includes endovascular thrombolysis/thrombectomy and stent placement, in addition to standard anticoagulation.

We report a case of MTS-related extensive iliofemoral DVT occurring in a middle age male who presented with seemingly unprovoked left iliofemoral vein thrombosis, complicated by pulmonary embolism.

## 2. Case Presentation

A 63-year-old Chinese gentleman was admitted with a 4-day history of progressive left lower limb swelling and pain. There was no history of trauma to the leg. He did not have any fever, chest pain, breathlessness, nor constitutional symptoms.

This was his first episode presented as such. He had a past medical history of hypertension and hyperlipidaemia, when he was on diet control with no chronic medications. There was no family history of DVT. He had no recent prolonged flights or immobilization, although he was working as a private car hire driver with ten-hour shifts. He was a never smoker.

**Figure 1:**
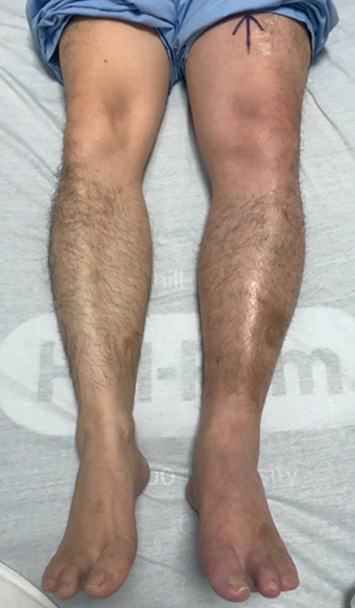
Unilateral oedematous swelling of left lower limb.

He was comfortable and afebrile with normal vitals and oxygen saturation. The cardiovascular and respiratory examinations were unremarkable. His left lower limb was warm and mildly tender, associated with swelling up to the midthigh measuring 4 cm more than the right (Figure 1). There was no evidence of stasis dermatitis, varicosities, or phlegmasia cerulean or alba dolens. His peripheral distal pulses were well felt.

**Figure 2:**
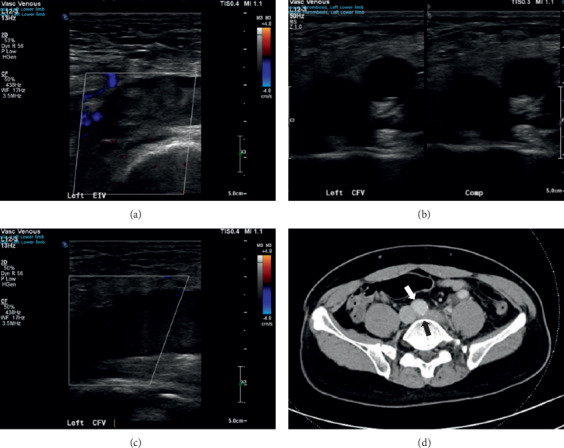
Ultrasound of left lower limb demonstrating (a) thrombosis involving the EIV, (b) failure of compressibility of CFV, and (c) no colour flow on duplex scan confirming the diagnosis of DVT. (d) CT of the patient showing the right CIA (white arrow) compressing the left CIV against the lumbar vertebrae (black arrow).

Initial blood investigations including full blood count, renal panel, and coagulation profile were normal; fasting glucose for diabetes screening was negative. Electrocardiogram showed sinus rhythm with no heart strain. Doppler compression ultrasonography of the left lower limb showed partial DVT extending from the external iliac (EIV), common femoral (CFV), to the popliteal veins (Figures 2(a)–2(c)).

In view of the extensive nature of the thrombosis, a contrasted CT of the thorax abdomen and pelvis was performed both to rule out an intrabdominal pathology as well as look for pulmonary complications in the same sitting. His CT eventually showed compression of the left common iliac vein (CIV) against the lumbar vertebrae by the overlying right common iliac artery (CIA), just caudal to its confluence with the right CIV (Figure 2(d)), with no definite filling defect identified in the IVC. In addition, filling defects in the right upper lobe and lower lobe pulmonary arterial lobar/segmental branches were noted, consistent with pulmonary embolism without evidence of right heart strain. In the absence of any suspicious pelvic mass, these features were suggestive of MTS. He was started on LMWH enoxaparin.

**Figure 3:**
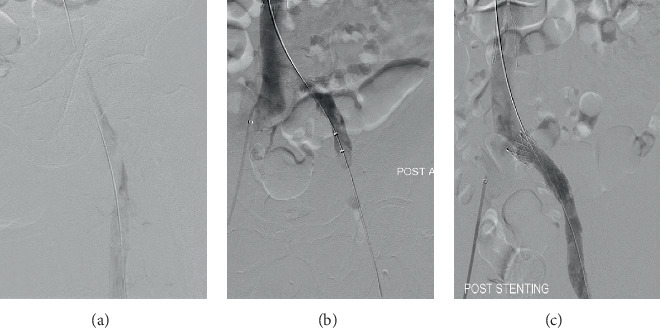
Intraoperative venogram (a) demonstrated extensive intravenous thrombus in the CFV and proximal SFV. Uninterrupted contrast flow through the CIV after (b) AngioJet pharmacomechanical thrombolysis/thrombectomy and (c) iliac vein stenting upon completion of the procedure.

During the operation, a temporary IVC filter was first inserted via a retrograde right femoral common femoral vein approach (Bard Denali) to mitigate the risk of massive pulmonary embolism and cardiovascular collapse during wire and device manipulation through the iliac thrombus. The left posterior tibial vein at the ankle was the initial vascular access site to allow full evaluation and thrombus removal of the entire femoropopliteal segment in addition to the iliac veins. Intraoperative venogram demonstrated extensive intravenous thrombus from the left CIV to proximal superficial femoral vein (SFV) (Figure 3(a)). This was treated with AngioJet pharmacomechanical thrombolysis/thrombectomy (Boston Scientific, Massachusetts, United States of America) using the ZelanteDVT thrombectomy catheter and a recombinant tissue plasminogen activator (alteplase 20 mg in 100 ml normal saline). Vascular access was then obtained via the left mid-distal femoral vein for diagnostic intravascular ultrasound (IVUS) which confirmed compression at the level of the CIV (cross-sectional area∼126 mm^2^) followed by iliac vein stenting using a 16 × 60 mm ABRE (Medtronic, Dublin, Ireland) bare metal stent. There was uninterrupted contrast flow through the left CIV upon completion of the procedure (Figures 3(b)–3(c)) with resolution of the compression and increased CIV cross-sectional area (∼198 mm^2^) on IVUS.

Postoperatively, he was placed on thromboembolic deterrent stockings and reviewed regularly by physiotherapists to prevent deconditioning. He was initially observed in the high dependency ward, with improvements of his lower limb swelling, and was subsequently transferred to the general ward where he remained well. His enoxaparin was switched to a Factor Xa inhibitor Apixaban (10 mg BD loading dose for 7 days followed by 5 mg BD maintenance dose), which he was discharged with to complete a total of 1 year duration. On his 1-month follow-up, the patient remained well with resolution of his lower limb swelling and pain. An ultrasound duplex iliac scan showed a patent left CIV stent with subacute to chronic venous thrombosis partially occluding the left CFV. He subsequently underwent an uneventful elective IVC filter retrieval, with plans for a 1-year repeat ultrasound and follow-up.

## 3. Discussion

MTS, also known as iliac vein compression syndrome (IVCS), was first described by May and Thurner in 1957 [1] and is an important anatomical risk factor for venous thrombosis. The pathogenesis of MTS-related DVT is likely to be a combination of vascular endothelial thickening, reduced vein diameter, and mechanical compression. Although this variant has been shown to be present in more than 20% of the population [1, 2], the reported clinical prevalence of MTS-related DVT has been low at only 2-3% of all lower limb DVTs [3, 4]. This may suggest an underestimation of its actual prevalence and is likely due to missed diagnosis, especially in patients with other more recognisable risk factors.

The most common clinical presentation is unilateral leg swelling due to acute DVT and is more common in women with the majority aged between 20 and 50 years old [5]. It can also present as chronic venous insufficiency and venous stasis with varicosities or ulceration. Other factors that may increase the likelihood that asymptomatic MTS will progress to symptomatic MTS include immobilization, contraceptive use, recent pregnancy, and previous intra-abdominal surgical interventions [6–8].

The treatment of MTS largely depends upon the presence of symptoms, their severity, and whether thrombosis is present. For symptomatic patients with iliofemoral DVT, as with our patient, treatment options include endovascular thrombolysis/thrombectomy and stent placement, in addition to standard anticoagulation. In observational studies and retrospective reviews of patients with MTS-related DVT, endovascular thrombolysis and/or stenting have consistently shown to be a safe and effective method for achieving high venous patency rates and provide relief of acute symptoms [4, 9–11]. In addition, though not stratified based on a diagnosis of MTS, systemic reviews of studies evaluating the treatment of acute iliofemoral thrombosis have shown that endovascular thrombolysis compared with anticoagulation alone is associated with higher iliofemoral patency rates and reduced risk for postthrombotic syndrome (PTS) [12–16].

The optimal duration of anticoagulation is unclear due to the lack of consensus on postintervention antithrombotic therapy for MTS patients [17]. It is recommended that therapeutic anticoagulation should be continued using similar dosing, monitoring, and duration per venous thromboembolism (VTE) guidelines [18]; direct oral anticoagulants (DOAC), including apixaban, rivaroxaban, edoxaban, and dabigatran, are effective alternatives to vitamin K antagonist but their use remain limited in specific populations with pregnancy, cancer, and renal insufficiency conditions. In patients who have had stent placement, anticoagulation for at least six months to prevent in-stent restenosis is recommended [5]. Following successful thrombolysis and stent placement, patients are also encouraged the use of sized-to-fit compression stockings for prevention of PTS as per acute DVT treatment [19, 20], assuming no contraindications including severe arterial insufficiency, overlying skin ulceration, or allergy to stockings material.

Our patient was diagnosed with MTS-related acute left iliofemoral DVT complicated by pulmonary embolism. His management included deployment of a temporary IVC filter, AngioJet pharmacomechanical thrombolysis/thrombectomy with iliac vein stent placement, and was discharged with apixaban with plans for repeat imaging to monitor stent patency.

## 4. Conclusion

May–Thurner syndrome is an important anatomical risk factor for venous thrombosis and is very often underdiagnosed in patients with DVT. Our case underscores the importance of having an index of suspicion in patients who present with seemingly unprovoked left iliofemoral vein thrombosis, including in males. The consideration and accurate diagnosis of MTS remains critical, as in addition to standard therapeutic anticoagulation, these patients would benefit from anatomical correction of the affected segment to maintain venous patency and prevent long-term complications.
